# Orange Peel Waste as Feedstock for the Production of Glycerol-Free Biodiesel by the Microalgae *Nannochloropsis oculata*

**DOI:** 10.3390/molecules28196846

**Published:** 2023-09-28

**Authors:** Giuseppe Tardiolo, Marco Sebastiano Nicolò, Carmelo Drago, Claudia Genovese, Giovanni Fava, Concetta Gugliandolo, Nicola D’Antona

**Affiliations:** 1 Department of Veterinary Sciences, University of Messina, Polo Universitario dell’Annunziata, Via Palatucci snc, 98168 Messina, Italy; gtardiolo@unime.it; 2 Department of Chemical, Biological, Pharmaceutical and Environmental Sciences, University of Messina, Viale Ferdinando Stagno d’Alcontres 31, 98166 Messina, Italy; concetta.gugliandolo@unime.it; 3 National Research Council, Institute of Biomolecular Chemistry, Via Paolo Gaifami 18, 95126 Catania, Italy; nicola.dantona@cnr.it; 4 National Research Council, Institute for Agricultural and Forest Systems in the Mediterranean, Via Empedocle 58, 95128 Catania, Italy; claudia.genovese@cnr.it; 5 Independent Researcher, 95126 Catania, Italy; giovanni.fava@hotmail.com

**Keywords:** *Nannochloropsis oculata*, microalgae cultivation, orange peel waste, acid hydrolysis, photoautotrophic, photoheterotrophic, bioconversion, lipid production and extraction, biodiesel

## Abstract

The bioconversion of agri-food waste into high-value products is gaining growing interest worldwide. Orange peel waste (OPW) is the main by-product of orange juice production and contains high levels of moisture and carbohydrates. In this study, the orange waste extract (OWE) obtained through acid hydrolysis of OPW was used as a substrate in the cultivation of the marine microalgae *Nannochloropsis oculata*. Photoheterotrophic (PH) and Photoautotrophic (PA) cultivations were performed in OWE medium and f/2 medium (obtained by supplementing OWE with macro- and micronutrients of f/2 medium), respectively, for 14 days. The biomass yields in PA and PH cultures were 390 mg L^−1^ and 450 mg L^−1^, while oil yields were 15% and 28%, respectively. The fatty acid (FA) profiles of PA cultures were mostly represented by saturated (43%) and monounsaturated (46%) FAs, whereas polyunsaturated FAs accounted for about 10% of the FAs. In PH cultures, FA profiles changed remarkably, with a strong increase in monounsaturated FAs (77.49%) and reduced levels of saturated (19.79%) and polyunsaturated (2.72%) FAs. Lipids obtained from PH cultures were simultaneously extracted and converted into glycerol-free biodiesel using an innovative microwave-assisted one-pot tandem protocol. FA methyl esters were then analyzed, and the absence of glycerol was confirmed. The FA profile was highly suitable for biodiesel production and the microwave-assisted one-pot tandem protocol was more effective than traditional extraction techniques. In conclusion, *N. oculata* used OWE photoheterotrophically, resulting in increased biomass and oil yield. Additionally, a more efficient procedure for simultaneous oil extraction and conversion into glycerol-free biodiesel is proposed.

## 1. Introduction

The bioconversion of agri-food waste into high-value products is gaining interest [1] as such wastes have increased as the global population and economy have grown [2,3]. The incorrect disposal of food waste has several negative impacts on the environment, including bad smells, gas emissions, and groundwater contamination [1]. Due to this, efforts have been made in recent years to explore the conversion of food waste into value-added products and to develop downstream processes of converting feedstocks into end-products [4,5]. 

Among the various agri-food products, oranges represent one of the top five fruit commodities that dominate the worldwide market [6]. In addition, orange juice is one of the most consumed beverages, and large amounts of this fruit are used in the manufacture of juice and marmalade [7]. Wastes produced during orange juice production include seeds, peels, and membrane residues [8], with a global estimated amount in the range of 15–25 million tons per year [6]. Among these wastes, orange peel waste (OPW) is the major constituent, accounting for approximately 44% of the fruit mass [9]. The exact chemical composition of oranges is affected by various factors [10]; however, the OPW usually contains 42.5% pectin, 16.9% soluble sugars, 10.5% hemicellulose, and 9.21% cellulose as the most relevant components [11]. The OPW is divided into organic and conventional. The first one is intended for producing feeds for organic livestock, while conventional pulp is exploited for biomethane production. Italian citrus production is estimated to be approximately 2.9 million tons per year, of which 12.6% is produced in Sicily, of which 55% is the corresponding overage waste (FaoStat, 2016). Herein, we have evaluated an alternative exploitation of conventional waste for biodiesel production, which could be considered complementary to anaerobic digestion in biomethane production. Traditional OPW recycling procedures such as landfilling, composting, pectin extraction, and animal feeding have many drawbacks and relatively high costs [12]. The disposal of OPW by bioconversion may be difficult because of the presence of several antimicrobial compounds (e.g., essential oils) and the high chemical complexity of the macromolecular content [8]. Thus, alternative biotechnological solutions are required for OPW recycling [12].

Several species of microalgae have been exploited as innovative sources in a variety of biotechnological applications to produce food, additive feed ingredients, cosmetics, biopharmaceuticals, nutraceuticals, pigments, and biofuels [13]. From a long-term sustainable energy perspective, microalgae are considered the most suitable raw material for third-generation biofuel production [14] since their lipid fraction can be extracted and converted into biodiesel [15]. In comparison to oleaginous plants, which are used as a traditional source of oil for biodiesel production, microalgae have several advantages such as high growth rate, high lipid content, ability to survive in adverse conditions, no competition for exploitation of agricultural land and food production, and the ability to sequester CO_2_ with higher efficiency [16].

Microalgae belonging to the genus *Nannochloropsis* are considered promising candidates for biofuel production, as they can potentially accumulate lipids up to 28.7% of their dry weight, or 65–70% of their dry weight under nutrient deprivation, resulting in lipid productivity ranging from 25.8 to 60.9 mg L^−1^ d^−1^ [17]. Such productivity can be compared with other important known commercial microalgal species [18] showing different growth rates and lipid content [17], for example, *Spirulina maxima* (8.6 mg lipid L^−1^ d^−1^), *Chlorella vulgaris* (9.2 mg lipid L^−1^ d^−1^), and *Dunaliella tertiolecta* (20.0 mg lipid L^−1^ d^−1^) [18]. The unusually flexible photosynthetic apparatus and the genome sequence of *Nannochloropsis* species make them suitable for various purposes [17]. Interestingly, a genetic transformation method utilizing homologous recombination in *Nannochloropsis* has been performed, suggesting potential perspectives in biofuels research [17]. *Nannochloropsis oculata* is characterized by high productivity and high fatty acid and triglyceride content, representing an interesting bio-source for nutraceutical and biodiesel feedstock [17,18]. The ability of this marine microalgae to grow in ponds of saline, brackish, and hypersaline water suggests the possibility of conducting studies to explore further potential applications of this species [17]. Besides photosynthetic mechanisms, several microalgae can use organic carbon sources such as sugars, organic acids, alcohols, and phenolics for growth [19]. For these reasons, microalgae have been investigated for their ability to dispose of and recycle various carbon-rich agro-industrial wastes. 

**Figure 1:**
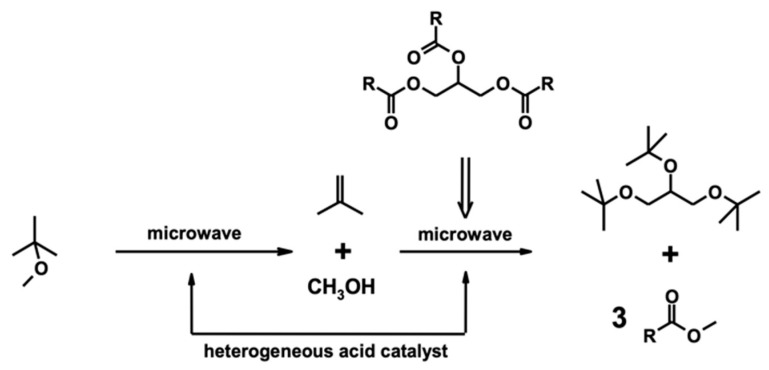
Decomposition of methyl-*tert*-butyl ether, transesterification of triglycerides, and glycerol etherification.

Current biodiesel production from microalgae is not economically viable due to the costs of biomass production, lipid extraction, and transesterification into fatty acid methyl esters (FAMEs) and purification of FAMEs from crude glycerol, which is a by-product of the transesterification process [20]. Regarding biodiesel production, most of the current industrial transesterification processes use alkaline chemical catalysts, which are inexpensive and result in high levels of conversion in short reaction times, even though they also produce glycerol as a by-product (about 10% of the produced biodiesel) that must be removed, with a consequential economic impact on the entire process [21]. Although it has been reported that crude glycerol can be a suitable substrate for microbial production of green chemicals [22] such as biosurfactants [23] and bioplastics [24,25], its bioconversion still has a considerable cost in terms of the energy required. In addition, even though glycerol is considered a key molecule in the preparation of many valuable organic compounds and several applications of glycerol and its derivatives are already well-known in the pharmaceutical, cosmetic, food, and beverage industries [26], the potential growth in biodiesel demand can threaten the downgrading of glycerol to industrial waste. The reintegration of glycerol into the biodiesel production chain as a fuel additive is a desirable solution that could make the whole process much more competitive and sustainable [27]. Among the various derivatives, glycerol ethers have the optimal chemical–physical properties for this purpose and are already effectively used as fuel additives [28]. Among all lipid extraction techniques, microwave-assisted extraction (MAE) holds promise for its efficiency, high oil yield with superior quality, and reduced extraction times. Microwave heating uses a non-contact heat source that can penetrate biomass, interact with polar molecules such as water, and evenly heat the entire sample [29]. While non-polar organic solvents such as hexane are transparent to microwave radiation, they are suitable for selectively extracting the lipidic fraction. This solvent transparency improves the efficiency of microwave interaction within cells and the destruction of the membrane and consequently improves lipid extraction. In our previous study [21], we reported a microwave-assisted one-pot transformation of vegetable oils into a mixture of FAMEs (biodiesel) and glycerol ether derivatives (biodiesel additives). The reaction requires the commercially available methyl *tert*-butyl ether (MTBE), which is catalyzed by a heterogeneous acid catalyst. In this process, MTBE is a single reagent suitable for both transesterification and transetherification (Figure 1). 

The present study evaluated the potential recycling of the acid hydrolysis extract of OPW, called Orange Waste Extract (OWE), as a low-cost carbon source for growth and oil production in the marine unicellular microalgae *Nannochloropsis oculata*. OWEs were prepared by adding different aliquots of OPW (20, 30, 50, and 100 g) to 100 mL of 3% H_2_SO_4_ in natural seawater (OWE 20, 30, 50, and 100, respectively) and then supplementing with standard macro- and micronutrients (OWE 20, 30, 50, and 100 media). The microalgae were able to use OWE media photoheterotrophically, in particular OWE50 medium, with increased biomass and oil yields. Moreover, lipids were extracted and converted into biodiesel using an innovative microwave-assisted one-pot tandem protocol that combines lipid extraction from lyophilized cells and subsequent transformation into glycerol-free biodiesel. The residue is still rich in biomolecules that can be exploited in other processes. Furthermore, the final exhausted waste could be used for energy production (such as pyrolysis). To the best of our knowledge, this is the first report in which (i) the use of OPW as an organic carbon substrate for growth and oil production by microalgae and (ii) a unique procedure for microalgal oil extraction and simultaneous conversion to glycerol-free biodiesel are described.

## 2. Results

### 2.1. OWE50 Yield after OPW Treatment and Quantitative Determination of Carbohydrates Using HPAE-PAD

After the acidic treatment of fresh OPW, the OWE50 yield was about 50% (*w*/*w*) with respect to the acidic extraction mixture (i.e., 1 kg of fresh OPW mixed with 2 L of 3% H_2_SO_4_ in natural seawater). To obtain 1 L of OWE50, about 0.625 kg of OPW was subjected to acid hydrolysis. The main carbohydrates detected in the OWE50 extract are listed in Table 1. The results show that the total sugar content was 21.35 g L^−1^, of which 68.3% was glucose, the most commonly used organic carbon source in microalgae cultivation. Fructose was present at 18.5%, followed by arabinose (10.4%), and rhamnose (2.8%).

### 2.2. Quantitative Determination of Ions in OWE50 Using Ion Chromatography

The main ions detected in OWE50 are listed in Table 2. Chloride (11,286.90 ± 0.73 mg L^−1^) and sodium (11,088.60 ± 2.34 mg L^−1^) were the most represented chemical species. The order of cations concentration was Na^+^ > Ca^2+^ > K^+^ > Mg^2+^, while that for anions was Cl^−^ > SO_4_^2−^ > PO_4_^3−^ > NO^3−^ > Br^−^. Comparing these values with the ionic content of the natural seawater used in OWE50, we observed an increase in potassium, calcium, and sulfate and the addition of nitrate and phosphate (useful for algae growth) attributable to the use of fresh OPW.

### 2.3. Growth Kinetics

**Figure 2:**
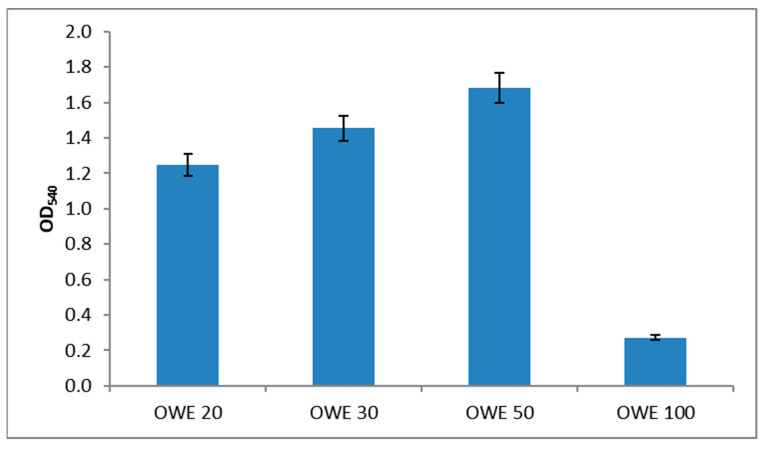
Growth of *N. oculata* in different OWE media after 14 days of incubation. The graph shows data from triplicate experiments (mean ± SD).

**Figure 3:**
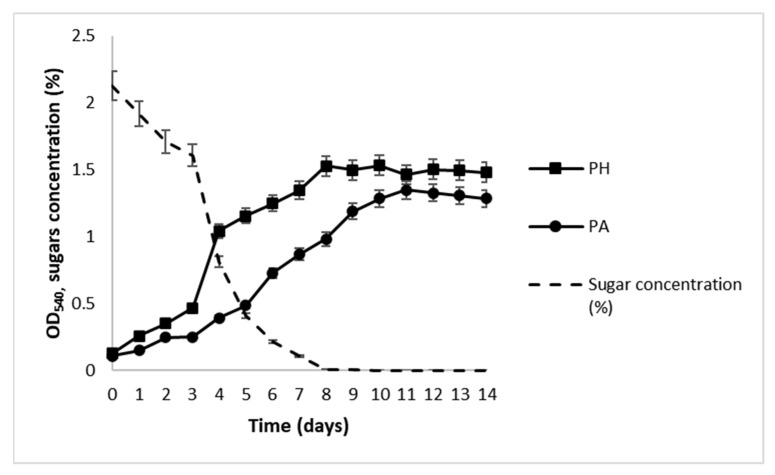
Growth of *N. oculata* under PA in f/2 medium or PH in OWE50 medium and total sugar concentration (dotted line). The graph shows data from triplicate experiments (mean ± SD).

The results of the growth of *N. oculata* in OWE20, 30, 50, and 100 culture media, obtained after acidic hydrolysis of different amounts of OPW (20, 30, 50, and 100 g; see Section 4 for details) are presented in Figure 2. Growth curves observed during photoautotrophic (PA) and photoheterotrophic (PH) batch processes are presented in Figure 3, and the values of growth kinetics variables, that is, specific growth rates (μ), biomass productivity (*P*), and biomass final yields, expressed as dry cell weight (DCW), are summarized in Table 3.

A comparison of the culture media containing different amounts of OWE showed that the OWE50 medium was the most suitable for PH cultivation; thus, this medium was used for all subsequent experiments.

During PA batch cultivation, cultures showed a lag phase in the first two days. Growth started and continued progressively until the 14th day, and then stopped (Figure 3). The specific growth rate (μ) was 0.210 d^−1^, while biomass productivity and yield were 27.85 mg L^−1^ day^−1^ and 390 mg L^−1^, respectively (Table 3). During PH batch cultivation, growth started after three days and reached the highest value after 8 days, then entered the stationary phase. In parallel, a drastic decrease in the concentration of free sugars was observed between the 3rd and 5th days and reached 0 after 8 days (Figure 3). For PH batch cultivation, the specific growth rate (μ) was 0.308 d^−1^, while biomass productivity and yield were 32.14 mg L^−1^ day^−1^ and 450 mg L^−1^, respectively (Table 3).

### 2.4. Lipid Yield and Fatty Acid Composition

Lipids from all cultures were extracted from 1 g of each dried sample, and the total lipid yield was expressed as a percentage of DCW. Fatty Acids (FAs) characterized using gas chromatography (GC) are reported in Table 4.

The lipid yield was 15% of DCW in PA cultures. Saturated FAs (SFAs, 42.90%) were mainly composed of palmitic acid (36.60%). Monounsaturated FAs (MUFAs) were present at a slightly higher percentage than SFAs (46.79%) and were mainly composed of palmitoleic (33.70%) and oleic (13.10%) acids. Polyunsaturated FAs (PUFAs) accounted for 10.31% of the FAs and were mainly represented by eicosapentaenoic acid. The lipid yield was 28% of DCW in PH cultures. SFAs represented 19.79% of the FAs, with palmitic acid (13.20%) being the most abundant. MUFAs were the predominant FA fraction (77.49%) and were principally composed of oleic (61.40%) and 10-octadecenoic (14.15%) acids. PUFAs accounted for 2.72% of the FAs and were exclusively represented by 9,11-octadecadienoic acid.

### 2.5. Conversion of Oil to Biodiesel Using Microwave-Assisted One-Pot Tandem Protocol and Analysis

For lipid extraction from *N. oculata*, different techniques for microalgal oil extraction were compared [30,31] using different solvents, reaction times, and temperatures of exposure (Table 5). Standard Soxhlet extraction gave the highest oil yield, followed by sonication-assisted extraction (SAE), with hexane as a solvent. SAE using MTBE/MeOH gave the worst performance. Interestingly, MAE efficiency was not influenced by the solvent used. We observed that oil yield using MTBE/MeOH (ratio of 9:1 *v*/*v*) was comparable to that obtained using hexane (Table 5). This result was particularly important as it allowed us to evaluate the possibility of directly converting microalgal oil into biodiesel using the microwave-assisted one-pot tandem protocol without any intermediate solvent exchange.

**Figure 4:**
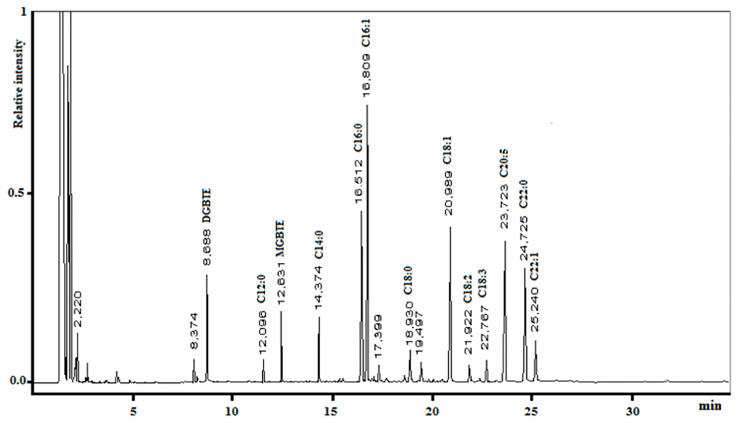
Gas chromatography profiles of FAMEs and glycerol ethers produced using MAE with MTBE/MeOH.

The microalgal oil obtained using MAE with MTBE/MeOH was poured into a microwave vessel, Amberlyst^®^-15 (Rohm and Haas Company, PA, USA) dry form was added as a catalyst, and the mixture was then irradiated (pre-set at 20 W, maximum temperature limit was set at 130 °C). After approximately three hours, we observed a complete transformation of triglycerides to the corresponding FAMEs and a mixture of *tert*-butyl glycerol derivatives; as expected, no free glycerol was detected (Figure 4).

## 3. Discussion

Microalgae products are used in the food and pharmaceutical industries as important sources of biomolecules such as PUFAs, proteins, vitamins, pigments, and so forth [13]. Microalgae also have applications in biofuel production as they are considered an alternative feedstock for third-generation biofuels [32,33]. The achievement of higher biomass and oil yields and growth using organic carbon sources has been described in several studies. Simple organic molecules such as acetate [34], glycerol, glucose [35], and ethanol [36], as well as more complex carbon sources such as aqueous extracts of sugarcane bagasse, or vegetable ground biomass such as corn stover, sugarcane bagasse, or switchgrass [37], are all suitable substrates for growing several *Nannochloropsis* species. 

Agroindustry wastes are currently being explored as potential organic substrates for the cultivation of various species of microalgae. The recycling of these by-products represents an important strategy for reducing environmental pollution and obtaining valuable products, according to the unwritten economical “*value from waste*” rule [13], improving the economic viability of microalgae-based industrial processes. This vision is the basis of the circular economy, defined as “a model of production and consumption, which involves sharing, leasing, reusing, repairing, refurbishing and recycling existing materials and products as long as possible” [38]. As a consequence, the biorefinery concept becomes a central model for driving the industry towards an eco-sustainable economy. A biorefinery is defined as “a facility (or network of facilities) that integrates biomass conversion processes and equipment to produce transportation biofuels, power, and chemicals from biomass” [39]. Biorefineries can use different kinds of feedstocks, including oil- and sugar-bearing crops (first-generation feedstocks), straw, bakery waste, and rotten fruits (second-generation feedstocks), or aquatic biomass such as microalgae (third-generation feedstocks). 

In this study, we aimed to verify whether OPW could be used as a source of simple sugars for microalgal growth and oil production. The results demonstrate that OWE50 represents a low-cost carbon source for PH cultivation of *N. oculata* and can be bio-converted to microalgal biomass and lipids. It is possible to set up an eco-friendly, integrated process for OPW exploitation as a low-cost carbon source or microalgae cultivation and oil production. Data indicate that the OWE50 medium contains a remarkable concentration of free sugars, mainly glucose, and ions, mainly sodium and chloride, with OPW contributing nitrate and phosphate necessary for microalgal growth. The OWE50 medium facilitated *N. oculata* growth and oil production, with yields and growth parameters, i.e., specific growth rate, biomass yield, and productivity, higher than those obtained during the PA batch process. The FAs profiles of lipids produced by the same microalgal species under different culture conditions may vary significantly [15]. Similar results were obtained for *N. oculata* using the adopted nutritional regimens. The amounts of SFAs and MUFAs were almost similar (42.90% and 46.79%, respectively) in PA cultures, while eicosapentaenoic acid (EPA) was the most represented PUFA, accounting for 7.02% of the FAs. On the contrary, in PH cultures, MUFAs increased up to 77.49%, with a strong reduction in SFAs (19.79%), while EPA was not detected. Moreover, although the increase in biomass and lipid yields from PH cultures are mild, it is worth noting that the relative percentages of MUFAs, SFAs, and PUFAs produced by *N. oculata* in OWE50 medium are excellent for biodiesel production, as they fully satisfy UNE-EN 14214 [40,41]. The proportions of SFAs, MUFAs, and PUFAs influence several critical parameters of biodiesel, including cetane number, iodine values, and cold flow properties. In particular, the high content of MUFAs, the low content of SFAs, and, overall PUFAs have been reported as a very good compromise for good quality biodiesel [42]. Furthermore, the MUFAs produced by PH cultures of *N. oculata* are represented by oleic and 10-octadecenoic acids, both containing 18 carbon atoms, which positively influence viscosity, cetane number, and heat of combustion [43]. 

Among the several extraction techniques evaluated (Table 5), MAE using a blend of MTBE/MeOH (ratio 9:1 *v*/*v*) was found to be a suitable combination for obtaining triglycerides from *N. oculata* biomass in terms of quality, time, and yield (almost 28%). The main advantage of this extraction technique includes reprocessing the mixture obtained directly in the microwave reactor for the transformation of triglycerides without any intermediate solvent exchange. Therefore, a complete transformation of the triglycerides to the corresponding FAMEs and a mixture of *tert*-butyl glycerol derivatives without any residual free glycerol is achieved. However, further pilot studies and variable optimization are necessary to evaluate the economic feasibility of the whole process. A similar research study was conducted by Park et al. [44] using orange peel extracts as an inorganic and organic nutrient source for the mixotrophic cultivation of the microalgae *Chlorella vulgaris*. Park et al. [44] reported increased production of biomass and FAMEs, suggesting that orange peel extract has the potential for use in the mixotrophic cultivation of microalgae for biodiesel production. Furthermore, several studies on species belonging to the genus *Nannochloropsis* have been conducted in recent years using different technological approaches [45,46,47,48,49,50,51].

## 4. Materials and Methods

### 4.1. Seawater Collection Site and Orange Peel Waste Treatment

The natural seawater used for microalgae cultivation was sampled from the east coast of Sicily (37°34′30″ N 15°10′29″ E); its ionic composition is reported in Table 2. Fresh OPW was obtained using Oranfresh^®^ S.r.l. (Catania, Italy). Before further processing, the OPW was ground in a food processor and transformed into small particles (<2 mm), and then subjected to heat-assisted acid hydrolysis. Briefly, different aliquots of OPW (20, 30, 50, and 100 g) were mixed with 100 mL of 3% H_2_SO_4_ in natural seawater and thermally treated in an autoclave at 121 °C for 30 min to hydrolyze polysaccharides and pectins. The solid fractions were separated via centrifugation at 5000× *g* for 20 min at 4 °C. The pH was then neutralized with 1 M sodium hydroxide, and the supernatants—orange waste extract (OWE) 20, OWE30, OWE50, and OWE100—were filtered at 0.22 µm to obtain a sterile and clear liquid. Finally, adequate aliquots of nutrient stock solutions used in the f/2 medium were added to all the OWEs to achieve the final millimolar concentrations present in the standard f/2 medium (nutrient-repleted OWEs) used to verify microalgae growth. The nutrient-repleted OWE50 (called OWE medium) showed the best result in terms of biomass yield (Figure 2) and was used in all the batch cultivations.

### 4.2. Quantitative Determination of Carbohydrates and Ions in OPW Hydrolysate

Quantification of the main carbohydrates in OWE50 was performed using high-performance anion-exchange chromatography with pulsed amperometric detection (HPAE-PAD, Thermo Scientific Dionex ICS3000, Sunnyvale, CA, USA). The sample, diluted to 1:2000 with deionized water and then filtered using a 0.20 µm nylon filter, was analyzed using a chromatography system equipped with a quaternary gradient inert pump, a pulsed amperometric detector, and an AS40 automated sampler. The separation was carried out using a Dionex CarboPac PA10 analytical column (250 × 4 mm i.d.) and a CarboPac PA10 guard column (50 × 4 mm i.d.). The acquisition of chromatograms was performed using the Chromeleon chromatography management system. All experiments were carried out at 30 °C under isocratic elution using 100 mM NaOH, at a flow rate of 0.8 mL min^−1^. Analyses were performed in triplicate; analyte quantifications were conducted using external standards (calibration curve range 0.5–10.0 mg L^−1^ for glucose and fructose, 0.2–2.0 mg L^−1^ for arabinose and rhamnose; R2 ≥ 0.9978) and results are reported in g L^−1^. The percentage of relative standard deviations of peak retention times was <0.8%.

Quantification of the main ions present in OWE50 was determined using ion chromatography (Thermo Scientific, Dionex ICS3000, Sunnyvale, CA, USA) with suppressed conductivity detection. Anions were separated using anion exchange column (Dionex IonPac AS22 (250 × 4 mm i.d.) with IonPac AG22 guard column (50 × 4 mm i.d.)) and cations using cation exchange column (Dionex IonPac CS12A (250 × 4 mm i.d.) with IonPac CG12A guard column (50 × 4 mm i.d.)). An aqueous solution containing 20 mM methanesulfonic acid was used to elute cations. The mobile phase containing 4.5 mM sodium carbonate and 1.4 mM sodium bicarbonate was used to elute anions. Flow rates of 1.0 mL min^−1^ and 1.2 mL min^−1^ were used to separate cations and anions, respectively. The column temperature was maintained at 30 °C during analysis. The sample was appropriately diluted (1:1000 and 1:500) with deionized water and filtered with a nylon syringe filter (0.45 µm). Analyses were performed in triplicate; analyte quantifications were conducted using external standards (range 0.5–100 mg L^−1^; R2 > 0.999) and results are reported in mg L^−1^ (Table 1). The percentage of relative standard deviations of peak retention times ranged from 0.7% to 2.1%.

### 4.3. Microalgae Stock Cultures, Batch Cultivations, and Photobioreactor Management

*Nannochloropsis oculata* (K-1281) was obtained from the Scandinavian Culture Collection of Algae and Protozoa. The strain was grown in f/2-Si medium [52,53]. Stock cultures were maintained at room temperature (20 °C ± 2) under continuous shaking and illuminated by white LED light at 40 µmol photons m^−2^ s^−1^ under a 12 h:12 h light:dark cycle. Batch cultivations were performed in an ePBR cylindrical bench photobioreactor (Phenometrics, Lansing, MI, USA) with a 450 mL working volume. The photobioreactor was operated according to the manufacturer’s recommendations. PA or PH batch cultivations were conducted in f/2 or OWE media, respectively, for 14 days. For each process, an inoculum of 10% *v*/*v* of stock cultures was used. Cultures were irradiated using white LED light (100 µmol photons m^−2^ s^−1^) under a 16 h:8 h light:dark cycle, aerated with filtered air (0.22 μm filter) bubbled into the cultures and continuously stirred (130 rpm) under a temperature of 20 °C (± 2). The daily growth of all the cultures was measured spectrophotometrically as optical density at 540 nm (OD_540_). Moreover, in PH batch cultivations, the concentrations of free sugars were evaluated daily using the methods reported by Dubois et al. [54]. The results are expressed as the means ± standard deviations (SDs) of the values obtained from three replicates and graphically plotted as growth curves in a Microsoft Excel sheet. The specific growth rate (μ) was calculated as follows:

$$\mu =\frac{Ln\left(\frac{N_{2}}{N_{1}}\right)}{t_{2}-t_{1}}$$

where ***N*_1_** and ***N*_2_** are biomass (OD_540_) at time 1 (***t*_1_**) and time 2 (***t*_2_**), respectively, and ***t*_1_** and ***t*_2_** are the days on which growth started and stopped, respectively [55]. Biomass productivity (***P***), expressed in mg L^−1^ d^−1^, was calculated according to the following equation:

$$P=\frac{N_{i}-N_{0}}{t_{i}-t_{0}}$$

where ***N_i_*** and ***N*_0_** are the biomass values (DCW, mg L^−1^) at time ***t_i_*** and ***t*_0_**, respectively [56].

### 4.4. Lipid Extraction and Yield

At the end of each cultivation process, the biomass was harvested via centrifugation at 6000× *g* for 10 min at 4 °C, resuspended in a minimal volume of sterile MilliQ water, kept at −80 °C for 15 h, and then lyophilized using an Alpha1-2LD Plus freeze drier (Martin Christ GmBH, Osterode, Germany). Lipids were extracted from the lyophilized biomass (1.0 g) with the techniques thereinafter described to compare their efficiencies.

### 4.5. Soxhlet, Sonication-Assisted, and Microwave-Assisted Extractions

For the Soxhlet extraction, 1.0 g of lyophilized cells contained in a cellulose thimble was put inside the Soxhlet chamber. Then, 300 mL of pure *n*-hexane was used to extract the lipids for 6 h at a rate of 10 refluxes h^−1^ and a temperature of 90 °C. *N*-hexane was then eliminated by rotavapor and the total lipid extract was weighted. 

Sonication-assisted extraction (SAE) of microalgal lipids was performed on lyophilized biomass (1.0 g) using hexane (20 mL) or methyl *tert*-butyl ether (MTBE) (20 mL) under cold sonication (35 Hz; 20 °C) condition through two sequential steps of 20 min each. The residual solid material from the first extraction step was removed by centrifugation and then re-extracted. The resulting organic solutions were combined and the solvent removed under reduced pressure. 

For the microwave-assisted extraction (MAE), an aliquot (500 mg) of lyophilized biomass was dissolved in a mixture of MTBE (4.5 mL) and methanol (MeOH, 0.5 mL) and poured into a microwave tube. The resulting mixture was irradiated in a microwave reactor, pre-set at 20 watts (maximum temperature limit was set at 90 °C) for 20 min. After completion, the residual solid material was removed by centrifugation and the solvent was then evaporated under reduced pressure to obtain the crude oil. Lipid yields obtained using the above-mentioned extraction techniques were calculated as the ratio between the amounts of extracted lipid and the biomass yield, measured as DCW, and expressed as percentages.

### 4.6. Lipid Transesterification Methods and Procedures

For lipid transesterification, microalgal lipid extracts underwent two different transesterification protocols. The first (standard procedure) resulted in glycerol as a by-product, while the second (microwave-assisted procedure) did not. For the standard lipid transesterification procedure, quantitative lipid transesterification was carried out according to the following standard procedure: a 0.5 N methanolic sodium methoxide solution (8 mL) was slowly added to a stirred solution of oil (0.2 g) in toluene (4 mL). The resulting mixture was heated at 50 °C and left under stirring for almost 10 min before glacial acetic acid (0.4 mL) and water (20 mL) were added. The resulting mixture was poured into a separatory funnel, diluted with hexane (10 mL), and then extracted; the organic layer was collected while the aqueous phase was back-extracted with hexane (3 × 10 mL). The collected organic phase was dried on anhydrous sodium sulfate, filtered, and the solvent removed under reduced pressure. The final crude product, which is a mixture of FAMEs, was taken up using a known volume of hexane, filtered through a 0.45 μm PTFE membrane filter, and then subjected to GC/MS-FID analysis. For the microwave-assisted lipid transesterification procedure, a sample of microalgal lipid extract (250 mg) was dissolved in MTBE (1.6 mL, d 0.744 mg mL^−1^; 24.5 mmol) in a microwave tube and Amberlyst^®^-15 dry form catalyst (30 mg) added to the resulting solution. The reaction mixture was irradiated in a microwave reactor pre-set at 20 watts (the maximum temperature limit was set at 130 °C) until complete conversion of the substrate (3 h). The transformation of triglycerides into corresponding FAMEs (reaction progress) was monitored via HPLC analysis; once completed, the GC analysis of the final mixture showed the FAME composition and the presence of both di- and mono-*tert*-butyl glycerol ethers.

### 4.7. HPLC Analysis and FA Profiles Determined Using GC Analysis 

HPLC analysis was conducted using a Varian 9010 instrument (Varian Inc., Palo Alto, CA, USA) equipped with an Alltech 3300 evaporative light scattering detector (ELSD) (BÜCHI Labortechnik, AG, Flawil, CH). A Luna C18 column (250 × 4.6 mm, 5 µm particle size) from Phenomenex (Torrance, CA, USA) was used for separating triglycerides and FAMEs. HPLC conditions were as follows: eluent A, MeOH; eluent B, CH_2_Cl_2_; gradient: 0–3 min (A-B/80:20), 3–18 min (A-B/30:70), 18–23 min (A-B/30:70); flow rate: 1 mL min^−1^. ELSD was set to a probe temperature of 40 °C and a gain of 16, and the nebulizer nitrogen gas was adjusted to 1.5 L min^−1^.

GC analyses were conducted using Shimadzu GC-17A (Shimadzu Corporation, Kyoto, Japan) equipped with a fused silica capillary column from J&W Scientific (INNOWAX, 30 m, 0.25 mm, 0.25 µm) (Agilent Technologies, Santa Clara, CA, USA); nitrogen was used as the carrier gas (flow rate of 1 mL min^−1^). GC conditions were as follows: the injector and detector temperatures were set at 250 and 280 °C, respectively; the aliquot of the reaction mixture was injected and analyzed using the following temp. prog.: 60 °C for 2 min, 60–200 °C at 10 °C min^−1^, 200–250 °C at 5 °C min^−1^, 250 °C for 5 min. Identification of different FAMEs and, eventually, the presence of *tert*-butyl glycerol ethers, was achieved by referring to the chromatograms of standard compounds.

### 4.8. Microwave-Assisted One-Pot Tandem Protocol for Lipid Extraction–Transesterification Process 

A sample of lyophilized microalgal biomass (1 g) was dissolved in a mixture of MTBE (4.5 mL) and MeOH (0.5 mL) and poured into a microwave tube. The resulting mixture was irradiated in a microwave reactor pre-set at 20 watts (the maximum temperature limit was set at 90 °C) for 20 min. After completion, the residual solid material was removed via centrifugation, the resulting solution was poured into a microwave tube, and Amberlyst^®^-15 dry form catalyst (30 mg) added. The reaction mixture was irradiated in a microwave reactor pre-set at 20 watts (the maximum temperature limit was set at 130 °C) until complete conversion of the substrate as monitored using HPLC analysis (3 h). GC analysis of the final mixture showed the FAME composition and the presence of both di- and mono-*tert*-butyl glycerol ethers. 

### 4.9. Statistical Analysis

Data from the different experimental groups were compared using one-way analysis of variance and the Tukey-b test for post hoc analysis. All statistical values were considered significant at a *p*-level of 0.05. Statistical analyses were performed using Instat version 2.10 for Microsoft Windows (GraphPad Software Inc., San Diego, CA, USA).

## 5. Conclusions

OPW is the most abundant waste generated during the production of orange juice. After heat-assisted acid hydrolysis, the extract obtained had a good content of free sugars (21.35 g L^−1^) and ions (15,694 mg L^−1^ of cations and 17,795 mg L^−1^ of anions), indicating that it is a suitable organic carbon source. In PH cultures, OWE50 medium supported microalgae growth and oil production. The experimental approach adopted in this study suggests that the use of OPW as a low-cost carbon source combined with the production of high-value-added compounds such as microalgal oils can be performed as a single integrated process. Interestingly, PH cultivation affected the fatty acid composition, acting on the global unsaturation degree of the mixture of the fatty acids. The oil obtained from photoautotrophic metabolism showed interesting levels of PUFAs, whereas that obtained from photoheterotrophic metabolism could be used for biodiesel production. Furthermore, we reported a versatile microwave-assisted one-pot tandem protocol for lipid extraction and transformation into glycerol-free biodiesel, using the same solvent media in both steps (28% yield). This result, while making the whole process very simple and practical to obtain biodiesel from microalgae, also offers the advantage of avoiding both biodiesel purification from crude glycerol and intermediate solvent exchange, which requires a distillation step, giving it a high added value in terms of cost, operation time, and sustainability. The absence of free glycerol in the final mixture makes the process advantageous as it allows the production of FAMEs without requiring steps to remove it, easy recovery and reuse of both the heterogeneous catalyst—through a simple filtration—and excess solvent—by simple distillation. Finally, the mixture obtained may be suitable for use directly in the energy chain as a biofuel. Further studies should be conducted to increase the yield of sugars extracted using standard and green technologies for fresh OPW treatment to set up economically viable, eco-friendly processes at a pre-industrial scale.

## Figures and Tables

**Table 1:** Carbohydrate content (g L^−1^) of OWE50.

N°	Retention Time (min)	Analyte	Concentration (g L^−1^)	% of Total Sugar
1	3.75	Rhamnose	0.60 ± 0.01	2.8 ± 0.05
2	4.30	Arabinose	2.22 ± 0.01	10.4 ± 0.04
3	5.27	Glucose	14.58 ± 0.37	68.3 ± 1.73
4	5.75	Fructose	3.95 ± 0.06	18.5 ± 0.29
		Total sugar analyzed	21.35 ± 0.41	100

Data are expressed as mean ± SD (n = 3).

**Table 2:** Ion content (mg L^−1^) of OWE50 and the natural seawater used in its preparation.

Analyte	Concentration in OWE50	Concentration in Natural Sea Water
Cations		
Sodium	11,088.60 ± 2.34	10,739.24 ± 3.42
Potassium	1483.80 ± 0.03	386.12 ± 0.08
Magnesium	1419.50 ± 0.22	1284.00 ± 0.32
Calcium	1702.40 ± 0.28	401.63 ± 0.15
Anions		
Chloride	11,286.90 ± 0.73	18,990.78 ± 1.67
Bromide	24.05 ± 0.01	54.23 ± 0.04
Nitrate	34.40 ± 0.04	2.10 ± 0.01
Sulphate	6377.50 ± 1.96	2660.42 ± 0.86
Phosphate	71.80 ± 0.01	0.07 ± 0.01

Data are expressed as mean ± SD (n = 3).

**Table 3:** Values of specific growth rate (*μ*), biomass productivity (*P*), and biomass yield (expressed as DCW) in *N. oculata* cultivated under PA and PH batch processes.

Growth Kinetic Parameters			
	PA	PH	Δ (%)
Specific growth rate (µ, day^−1^)	0.210 ± 0.016	0.308 ± 0.011	+46.67
Biomass productivity (*P*, mg L^−1^ day^−1^)	27.85 ± 0.48	32.14 ± 0.62	+15.40
Biomass yield (DCW) (mg L^−1^)	390 ± 6.7	450 ± 8.6	+15.38

Δ = difference between PH and PA values, expressed as a percentage. The values are data from triplicate experiments (mean ± SD).

**Table 4:** Fatty acid composition of the microalgal lipids from PA and PH batch processes.

Fatty Acids	%	
	PA	PH
3-Hydroxydecanoic (C10:0)	n.d.	0.55 ± 0.01
Lauric (C12:0)	1.20 ± 0.01	0.95 ± 0.01
Myristic (C14:0)	3.70 ± 0.04	1.95 ± 0.03
Pentadecanoic (C15:0)	0.90 ± 0.01	0.15 ± 0.005
Palmitic (C16:0)	36.60 ± 0.185	13.20 ± 0.19
Palmitoleic (C16:1)	33.70 ± 0.16	1.95 ± 0.021
Stearic (C18:0)	2.00 ± 0.019	3.05 ± 0.027
Oleic (C18:1)	13.10 ± 0.11	61.40 ± 0.55
10-Octadecenoic (18:1)	n.d.	14.15 ± 0.11
Linoleic (C18:2)	2.00 ± 0.017	n.d.
9,11-Octadecadienoic (C18:2)	2.10 ± 0.014	2.72 ± 0.019
Arachidonic (C20:4)	1.30 ± 0.001	n.d.
Eicosapentaenoic (C20:5)	7.02 ± 0.045	n.d.
Saturated Fatty Acids	42.90 ± 0.38	19.79 ± 0.16
Monounsaturated Fatty Acids	46.79 ± 0.37	77.49 ± 0.68
Polyunsaturated Fatty Acids	10.31 ± 0.14	2.72 ± 0.019

The values, expressed as percentages, are data from triplicate experiments (mean ± SD) (n.d. = not detected).

**Table 5:** Oil yields obtained from *N. oculata* using different lipid extraction techniques.

					% *w*/*w*
Entry	Lipid Extraction Technique	Time (min)	Temperature/Power	Solvent	Oil Yield (%)
1	Soxhlet	360	90 °C	Hexane	35 ± 0.95
2	SAE	20	4 °C	Hexane	32 ± 0.63
3	SAE	20	4 °C	MTBE/MeOH	24 ± 0.41
4	MAE	20	90 °C/20 W	Hexane	30 ± 0.58
5	MAE	20	90 °C/20 W	MTBE/MeOH	28 ± 0.43

SAE = sonication-assisted extraction; MAE = microwave-assisted extraction; MTBE = methyl *tert*-butyl ether; MeOH = methanol. The values, expressed as percentages, are data from triplicate experiments (mean ± SD).

## Data Availability

Supporting data for this study are available from the corresponding authors upon reasonable request.

## Sample Availability

Samples of the compounds obtained in this study are available from the corresponding authors upon reasonable request.
